# Risk factors and prognosis in very low birth weight infants treated for hypotension during the first postnatal week from the Korean Neonatal Network

**DOI:** 10.1371/journal.pone.0258328

**Published:** 2021-10-14

**Authors:** Young Hwa Song, Jin A. Lee, Byung Min Choi, Jae Woo Lim

**Affiliations:** 1 Department of Pediatrics, Konyang University College of Medicine, Daejeon, Korea; 2 Department of Pediatrics, Seoul National University College of Medicine, Seoul, Korea; 3 Department of Pediatrics, Seoul National University-Seoul metropolitan government Boramae Medical Center, Seoul, Korea; 4 Department of Pediatrics, Korea University College of Medicine, Seoul, Korea; Kobe University Graduate School of Medicine School of Medicine, JAPAN

## Abstract

Hypotension in the early stages of life appears in 20% of very low birth weight (VLBW) infants. The gestational age and birth weight are the risk factors highly related to the postnatal hypotension. Other risk factors slightly differ between different studies. So, we evaluated the risk factors and prognosis that are associated with infants treated with hypotension in the early stages of life, after excluding the influences of gestational age and small for gestational age (SGA). VLBW infants registered in the Korean Neonatal Network between 2013 and 2015 treated for hypotension within a week after their birth were selected as study subjects. The rest were used as a control group. Risk factors and the prevalence of severe complications, including mortality, were investigated and compared after matching for gestational age and SGA. The treatment rate for hypotension within the first postnatal week was inversely related to decreasing gestational ages and birth weights. In particular, 63.4% of preterm infants born at ≤ 24 weeks’ gestation and 66.9% of those with a birth weight < 500 g were treated for hypotension within a week of birth. Regression analysis after matching showed that 1-minute Apgar score, neonatal cardiac massage or epinephrine administration, symptomatic patent ductus arteriosus, early onset sepsis, and chorioamnionitis were significantly associated with hypotension. In the hypotension group, mortality, grade 3 or higher intraventricular hemorrhage, periventricular leukomalacia, and moderate to severe bronchopulmonary dysplasia rates were significantly higher after the matching for gestational age and SGA. Hypotension during the first postnatal week is very closely related to the prematurity and the condition of the infant shortly after birth. Regular prenatal care including careful monitoring and appropriate neonatal resuscitation are very crucial to decrease the risk of hypotension in the early stages of life.

## Introduction

It is known that hypotension occurs in about 20% of very low birth weight (VLBW) infants within 48 hours after birth [1]. Immediately after birth, preterm infants have reduced myocardial contractility, increased systemic vascular resistance, and steal syndrome caused by patent ductus arteriosus (PDA) [1,2]. Neonatal hypotension has been reported to recover spontaneously over time after birth; therefore, there has been a recent trend toward “permissive hypotension” treatment rather than active treatment [3,4]. However, depending on the gestational age, birth weight, and perinatal factors, hypotension requiring active treatment still occurs, and various studies have been conducted to identify the risk factors for hypotension requiring treatment [5–11].

Neonatal hypotension requiring treatment increases with younger gestational age or with smaller birth weight. A large-scale study of VLBW infants born in 14 institutions in the United States confirmed that 93% of preterm infants born at 23 weeks and 73% of preterm infants born at 27 weeks were treated for hypotension in the neonatal period [5]. Various perinatal factors, such as the Apgar score, antenatal steroid administration, and chorioamnionitis, are known to be associated with neonatal hypotension, but the reported associations have differed depending on the study [7,8,12–14].

Preterm infants treated for hypotension within the first postnatal week are known to have a high mortality rate and poor neurological prognosis, including delayed motor development and hearing loss [15]. Furthermore, high mortality and levels 3 and 4 intraventricular hemorrhage (IVH) have been commonly reported in infants with hypotension [16]. The major diseases and outcomes of preterm infants are known to be mainly associated with gestational age and birth weight, and hypotension occurring within the first postnatal week also occurs in inverse proportion to gestational age and birth weight [5]. Additionally, small for gestational age (SGA) was found to have a significant effect on the prognosis of preterm infants in previous studies [17].

Research on VLBW infants in South Korea reported that the rate of hypotension treatment within the first postnatal week was 57.2%, but these existing studies were often limited to cases in specific institutions; thus, these study populations may not represent the entirety of VLBW infants in South Korea [4,18].

Therefore, to accurately compare the risk factors and prognosis of the VLBW infants who required treatment for hypotension within a week after their birth, studies including larger number of subjects and excluding the effects of gestational age or SGA are needed. This study was conducted using big data collected by the Korean Neonatal Network (KNN) and the risk factors and prognosis were analyzed after the frequency matching to gestational age and SGA.

## Materials and methods

Data were prospectively collected from 67 neonatal intensive care units (NICUs) registered in the KNN. The KNN is a nationwide, multicenter, prospective, web-based cohort registry system for VLBW infants with a birth weight < 1,500 g, and it includes > 80% of VLBW infants in South Korea. This registry was established to improve data collection systems and to study various factors associated with the mortality and morbidity of VLBW infants in South Korea [19,20].

The KNN registry was approved by the institutional review board (IRB) at each participating hospital, including the Konyang University Hospital’s IRB (IRB number 2013-01-035). Written informed consent was obtained from the parents of all infants at enrollment by all the NICUs participating in the KNN. All methods were carried out in accordance with the IRB-approved protocol and in compliance with relevant guidelines and regulations. Each patient’s identification code was anonymized to protect the individual’s privacy. This study was approved by the KNN data management committee and the Konyang University Hospital IRB (IRB number 2017-07-015).

Among 5,844 VLBW infants admitted to the NICU from January 2013 to December 2015, infants with a gestational age < 22 weeks or ≥ 31 weeks, with a birth weight < 300 g, with fatal congenital anomalies or death due to congenital anomalies, who were transferred to another hospital, and who had been hospitalized for 365 days or longer were excluded. The remaining 4,191 patients were studied. Of these, 1,256 patients who received medication due to hypotension within a week of birth at the KNN participating hospitals were defined as the hypotension group, and 2,935 patients who did not receive treatment for hypotension or did not show symptoms of hypotension were defined as the control group. In this study, hypotension in preterm infants was defined as a mean arterial pressure (MAP) below that for the gestational age or a MAP < 30 mmHg. Treatment of hypotension was defined as the administration of medications including inotropic agents (dopamine, dobutamine, and epinephrine), hydrocortisone, and other medications such as vasopressin. There was only one case where vasopressin was administered. Infants who do not receive the aforementioned medication or received only volume expander treatment, such as normal saline, were not included in the hypotension group.

The general characteristics of the two groups were compared. The association between various risk factors of hypotension treated within a week after birth was examined. Neonatal factors compared included the gestational age, birth weight, SGA status, sex, hospital birth status, Apgar score at 1 and 5 minutes, need for cardiopulmonary resuscitation at birth (oxygen administration, positive pressure ventilation, tracheal intubation, cardiac massage, and epinephrine administration), neonatal body temperature, neonatal pH at the time of admission to the NICU, symptomatic PDA, and early onset sepsis (EOS). For SGA, infants were divided into severe SGA (< 3^rd^ percentile) or mild SGA (3–9^th^ percentile) based on the Fenton growth chart for preterm infants revised in 2013, and the rate of hypotension was compared according to the severity of SGA [21]. Symptomatic PDA was defined as confirmation of a large left-to-right ductal flow via color flow Doppler echocardiography with two or more of the following: (1) systolic murmur or continuous murmur, (2) bounding pulse or hyperactive precordial pulsation, (3) difficulty maintaining blood pressure (e.g., hypotension unresponsive to fluid therapy or dopamine; hypotension was defined as a blood pressure below the lower limit of the normal arterial blood pressure for the corrected age), (4) exacerbation of respiratory condition, and (5) evidence of chest radiographic findings (e.g., cardiomegaly [cardiothoracic ratio > 60%] accompanied by pulmonary congestion and increased pulmonary blood flow). EOS was defined as the diagnosis of sepsis within 7 days of birth, and sepsis was diagnosed based on bacterial identification by blood culture and the need for systemic antibiotic therapy for 5 days or longer. The maternal factors compared included the maternal age, education, multiple births, artificial insemination, delivery method, prenatal steroid administration and completion, chorioamnionitis, premature rupture of membranes, and pregnancy complications.

Multiple logistic regression analyses were performed on the association between the selected risk factors found to be significant in simple comparisons and selected factors reported to be significant in previous studies. In addition, the two groups were compared in terms of the rates of mortality, grade 3 or above IVH, cystic periventricular leukomalacia (PVL), moderate to severe bronchopulmonary dysplasia (BPD), and stage 3 or higher retinopathy of prematurity (ROP). The most severe stage of IVH on all brain sonography findings until the first discharge was recorded, and IVH staging was performed according to classification [22]. BPD was defined as the need for oxygen at 36 weeks’ postmenstrual age (PMA). Moderate BPD was defined as the need for oxygen for > 28 days plus < 30% oxygen at 36 weeks’ PMA using the National Institute of Child Health and Human Development (NICHD) Workshop severity-based diagnostic criteria. Severe BPD was defined as the need for oxygen for > 28 days plus ≥ 30% oxygen and/or positive pressure at 36 weeks’ PMA according to the NICHD criteria. Statistical comparison of morbidity was performed excluding cases when the diagnosis of each disease was not definitive.

**Fig 1:**
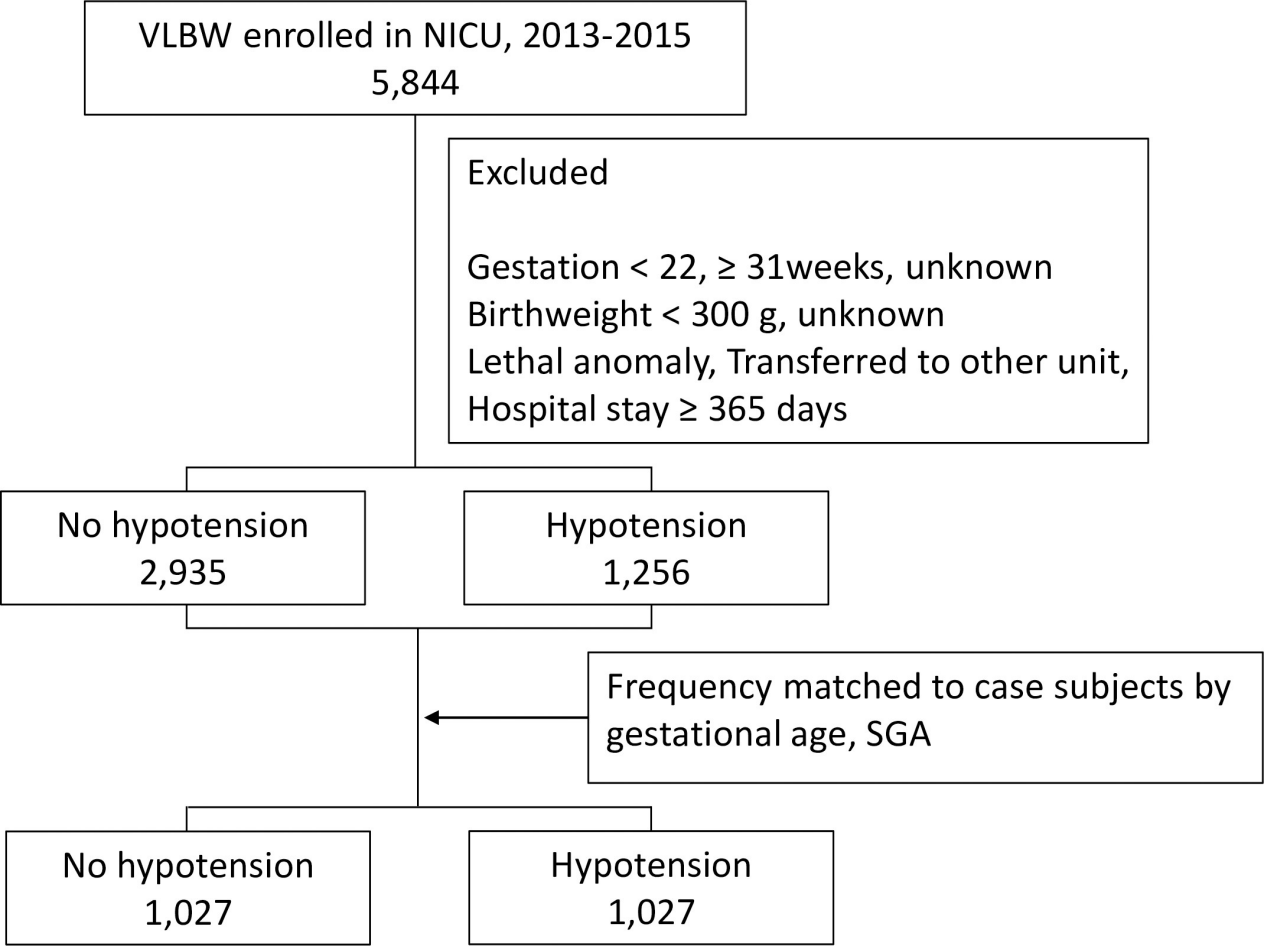
Flow-chart identifying the study population.

To compare the risk factors and prognosis between two groups clearly, frequency matching was performed according to the gestational age and SGA status. After matching, the subject group included 2,054 infants, with 1,027 infants in each group (Fig 1).

Statistical Package for the Social Sciences version 24 (SPSS, IBM Corp., Armonk, NY, USA) was used for the statistical analysis. Continuous variables are represented as mean and standard deviation and were compared using the Student t-test. Categorical variables were compared using the chi-squared test. When there were < 30 subjects for comparison, the Mann-Whitney test and Fisher exact test were used as non-parametric tests for continuous and categorical variables, respectively. Logistic regression analysis was used to analyze risk factors associated with hypotension. Poisson regression analysis was used to analyze the annual trend of drug for hypotension. *P* < 0.05 was considered statistically significant.

## Results

### Characteristics of the study population

The mean gestational age of the total patient group was 27.2 weeks, 25.9 weeks in the hypotension group, and 27.7 weeks in the control group. The mean birth weight was 1,016 g in the total group, 864 g in the hypotension group, and 1,081 g in the control group. The rate of hypotension was significantly higher in the SGA < 3^rd^ percentile than in the SGA between the 3^rd^ and 9^th^ percentiles or the appropriate for gestational age (AGA) or above. The sex ratio did not differ between the groups. After matching for gestational age and SGA, the mean gestational age of the two groups was 26.3 weeks, and the birth weights were 910 g in the hypotension group and 923 g in the control group, showing no statistical difference. There was no statistically significant difference in the maternal age, percentage of in vitro fertilization, and birth by cesarean section between the groups both before and after matching (Table 1).

**Table 1:** Demographic characteristics of the populations.

					Matched populations^a^		
Parameter		No hypotension (n = 2935)	Hypotension (n = 1256)	Total (n = 4191)	No hypotension (n = 1027)	Hypotension (n = 1027)	Total (n = 2054)
		n (%)	n (%)	n (%)	n (%)	n (%)	n (%)
**Neonatal**							
Gestational age (weeks, mean±SD)		27.7±1.9	25.9±2.1*	27.2±2.1	26.3±2.0	26.3±2.0	26.3±2.0
Gestational age,	22–24 weeks	200 (36.6)	346 (63.4)*	534 (100.0)	192 (50.1)	192 (49.9)	384 (100.0)
	25–27 weeks	964 (62.1)	588 (37.9)*	1552 (100.0)	538 (50.0)	538 (50.0)	1076 (100.0)
	28–30 weeks	1771 (84.6)	322 (15.4)*	2093 (100.0)	297 (50.1)	297 (49.9)	594 (100.0)
Birth weight (g, mean±SD)		1081±259	864±268*	1016±280	923±251	910±254	916±252
Birth weight,	< 500 g	39 (33.1)	79 (66.9)*	118 (100.0)	34 (50.0)	34 (50.0)	68 (100.0)
	500–999 g	1034 (56.3)	801 (43.7)*	1835 (100.0)	609 (48.9)	639 (51.1)	1248 (100.0)
	1000–1499 g	1862 (83.2)	376 (16.8)*	2238 (100.0)	384 (52.1)	354 (47.9)	738 (100.0)
Small for gestational age < 3^rd^ percentile		54 (42.9)	72 (57.1)*	126 (100.0)	35 (50.0)	35 (50.0)	70 (100.0)
	3^–^9^th^ percentile	160 (66.1)	82 (33.9)	242 (100.0)	56 (50.0)	56 (50.0)	112 (100.0)
Appropriate for gestational age or above		2710 (71.2)	1094 (28.8)*	3804 (100.0)	928 (50.1)	928 (49.9)	1856 (100.0)
Sex ratio		51.0:49.0	52.8:47.2	51.5:48.5	51.5:48.5	53.3:46.7	52.4:47.6
**Maternal**							
Age (years, mean±SD)		32.8±4.2	32.8±4.3	32.8±4.2	32.9±4.1	32.9±4.3	32.9±4.2
Pregnancy process, IVF		638 (21.7)	290 (23.1)	928 (22.1)	232 (22.6)	233 (22.7)	465 (22.6)
Delivery by cesarean section		2155 (73.4)	909 (72.4)	3064 (73.1)	742 (72.2)	766 (74.6)	1508 (73.4)

SD, standard deviation; IVF, in vitro fertilization.

^a^Results from the data with frequency matching by gestational age and small for gestational age.

*P < 0.01,

**P < 0.05.

### Comparison of neonatal and maternal risk factors of hypotension during the first postnatal week

The mean scores of the 1- and 5-minute Apgar scores were significantly lower in the hypotension group than in the control group, and the rates of the 1- and 5-minute Apgar scores ≤ 3 were significantly higher in the hypotension group than in the control group. The hypotension group showed significantly higher rates of cardiac massage, epinephrine injection, body temperature below 36°C, and pH below 7.20 at the time of admission to the NICU than the control group. Even after gestational age and SGA were matched, the mean 1- and 5-minute Apgar scores in the hypotension group were significantly lower than those in the control group, and the rates of the 1- and 5-minute Apgar scores ≤ 3 were significantly higher in the hypotension group than in the control group. After matching, the rates of neonatal cardiac massage and a neonatal pH < 7.20 were significantly higher in the hypotension group than in the control group.

The clinical risk index for babies-II (CRIB-II) score is not a risk factor but is used as a predictor for mortality; thus, we compared the differences in the index between the two groups. The mean CRIB-II score in the hypotension group were significantly higher than those in the control group. In a previous study, the cutoff point for prediction of mortality using the CRIB-II score has been reported as 11 points [23]. The rate of CRIB-II scores ≥ 11 points in the hypotension group was 37.3%, indicating a significantly higher value than that in the control group (10.6%). After gestational age and SGA were matched, these significances were disappeared.

The rate of symptomatic PDA was significantly higher in the hypotension group (50.3%) than in the control group (30.7%), and the results were consistent even after matching. The rate of EOS was statistically significantly higher in the hypotension group (7.6%) than in the control group (3.8%), and the results were consistent even after matching.

The rates of completion and unuse of antenatal steroid administration were significantly different between the control group (47.1% and 19.0%, respectively) and hypotension group (43.5% and 24.3%, respectively), and incomplete use of antenatal steroid was not significantly different between the groups. The incidence of maternal polyhydramnios was significantly higher in the hypotension group (3.4%) than in the control group (1.3%). Maternal diabetes mellitus was significantly lower in hypotension group (6.9%) than in the control group (8.8%). There were no statistically significant differences in multiple births, premature rupture of membranes, chorioamnionitis, and maternal hypertension. Even after matching for gestational age and SGA, the rates of completion and unuse of antenatal steroid administration were statistically significant between the groups; the maternal polyhydramnios rate was significantly higher in the hypotension group than in the control group; and the multiple birth rate was significantly higher in the hypotension group than in the control group. Maternal chorioamnionitis was significantly less common in the hypotension group compared to the control group after matching (Table 2).

**Table 2:** Risk factors of treated hypotension in VLBW infants during the first postnatal week.

				Matched population^a^		
Parameter	No hypotension (n = 2935)	Hypotension (n = 1256)	Total (n = 4191)	No hypotension (n = 1027)	Hypotension (n = 1027)	Total (n = 2054)
	n (%)	n (%)	n (%)	n (%)	n (%)	n (%)
**Neonatal**						
Apgar score at 1 min (mean±SD)	4.6±1.9	3.3±1.9*	4.2±2.0	4.1±1.8	3.5±1.9*	3.8±1.9
Apgar score at 1 min, 0–3	828 (28.3)	692 (55.6)*	1520 (36.5)	399 (39.0)	527 (51.8)*	926 (45.4)
Apgar score at 5 min (mean±SD)	6.9±1.7	5.7±2.0*	6.5±1.9	6.4±1.8	5.8±2.0*	6.1±1.9
Apgar score at 5 min, 0–3	135 (4.6)	194 (15.6)*	329 (7.9)	81 (7.9)	138 (13.6)*	219 (10.7)
Cardiac massage	87 (3.2)	149 (12.3)*	236 (6.0)	44 (4.4)	114 (11.6)*	158 (7.9)
Epinephrine administration	56 (2.1)	119 (9.8)*	175 (4.4)	34 (3.4)	89 (9.0)*	123 (6.2)
Initial BT, < 36°C	666 (23.5)	398 (33.8)*	1064 (26.5)	295 (30.2)	293 (30.2)	588 (30.2)
Initial pH, < 7.20	435 (19.2)	269 (30.7)*	704 (22.4)	184 (23.2)	201 (28.3)**	385 (25.6)
CRIB-II score (mean±SD)	6.5±2.9	9.4±3.2	7.3±3.2	3.0±0.1	3.0±0.1	3.0±0.1
CRIB-II score, ≥ 11 points	312 (10.6)	469 (37.3)*	781 (18.6)	215 (27.8)	203 (29.4)	418 (28.6)
Symptomatic PDA	887 (30.2)	634 (50.5)*	1521 (36.3)	389 (38.8)	516 (50.2)*	905 (44.6)
Early onset sepsis	111 (3.8)	101 (8.0)*	212 (5.1)	49 (4.8)	79 (7.7)*	128 (6.2)
**Maternal**						
Antenatal steroid	2889 (98.4)	1219 (97.1)	4108 (98.0)	1011 (98.4)	999 (97.3)	2010 (97.9)
None	548 (19.0)	296 (24.3)*	844 (20.5)	190 (18.8)	240 (24.0)*	430 (21.4)
Incomplete	981 (34.0)	393 (32.2)	1374 (33.4)	372 (36.8)	329 (32.9)	701 (34.9)
Complete	1360 (47.1)	530 (43.5)*	1890 (46.0)	449 (44.4)	430 (43.0)*	879 (43.7)
Polyhydramnios	36 (1.3)	38 (3.4)*	74 (1.9)	11 (1.2)	27 (2.9)*	38 (2.1)
Oligohydramnios	352 (13.2)	158 (14.1)	510 (13.4)	134 (14.3)	112 (12.2)	246 (13.3)
Multiple birth	982 (33.5)	456 (36.3)	1438 (34.3)	317 (30.9)	365 (35.5)**	682 (33.2)
PROM	1186 (40.7)	505 (40.6)	1691 (40.6)	419 (41.1)	416 (40.8)	835 (41.0)
Chorioamnionitis	935 (37.9)	408 (39.7)	1343 (38.4)	392 (45.1)	319 (38.2)*	711 (41.7)
DM^b^	258 (8.8)	87 (6.9)**	345 (8.2)	69 (6.7)	81 (7.9)	150 (7.3)
HTN^c^	447 (15.2)	168 (13.4)	615 (14.7)	128 (12.5)	135 (13.1)	263 (12.8)

SD, standard deviation; BT, body temperature; CRIB, critical risk index for babies; PDA, patent ductus arteriosus; PROM, premature rupture of membrane; DM, diabetes mellitus; HTN, hypertension; VLBW, very low birth weight.

^a^Results from the data with frequency matching by gestation and small for gestational age.

^b^DM included gestational and overt diabetes mellitus.

^c^HTN included pregnancy-induced hypertension and chronic hypertension.

*P < 0.01;

**P < 0.05.

Among the risk factors, the birth weight, SGA status, Apgar score, neonatal resuscitation (cardiac massage or epinephrine administration), neonatal body temperature, neonatal pH, symptomatic PDA, EOS, prenatal steroid administration, amniotic fluid volume, chorioamnionitis, and multiple birth status were identified as factors associated with treatment for hypotension within the first postnatal week. Regression analysis demonstrated a significant relationship between a lower birth weight and 1-minute Apgar score ≤ 3 and a higher incidence of hypotension. Additionally, in infants who underwent neonatal resuscitation, had pH at admission < 7.20, were diagnosed with symptomatic PDA or EOS, whose mother had polyhydramnios, and were multiple births, the rate of hypotension was high. However, in the logistic regression analysis after matching, the incidence of hypotension was significantly higher in infants when the 1-minute Apgar score was ≤ 3, when neonatal resuscitation was performed, and when infants were diagnosed with symptomatic PDA or EOS. The rate of hypotension was significantly lower in infants with mothers with chorioamnionitis than in those without (Table 3).

**Table 3:** Multivariate logistic regression model for risk factors.

Risk factors		Matched population^a^
	Adjusted OR (95% CI)	Adjusted OR (95% CI)
Birth weight		
< 500 g	6.67 (3.15–14.13)	0.91 (0.42–1.99)
500–999 g	3.07 (2.45–3.84)	0.98 (0.75–1.29)
1000–1499 g	1.00	1.00
SGA		
< 3^rd^ percentile	1.28 (0.68–2.40)	-
3-9^th^ percentile	0.81 (0.54–1.21)	-
AGA or above	1.00	-
Apgar score 1 min, 0–3		
Yes	2.18 (1.75–2.70)	1.84 (1.41–2.39)
No	1.00	1.00
Neonatal resuscitation^b^		
Yes	3.21 (2.08–4.95)	2.64 (1.51–4.63)
No	1.00	1.00
Body temperature at admission		
< 36°C	0.94 (0.75–1.17)	0.78 (0.59–1.03)
≥ 36°C	1.00	1.00
Initial pH at admission		
< 7.20	1.46 (1.15–1.84)	1.30 (0.97–1.74)
≥ 7.20	1.00	1.00
Symptomatic PDA		
Yes	1.87 (1.52–2.29)	1.66 (1.29–2.14)
No	1.00	1.00
Early onset sepsis		
Yes	2.14 (1.44–3.16)	2.00 (1.23–3.26)
No	1.00	1.00
Antenatal steroid		
None	1.19 (0.90–1.57)	1.08 (0.77–1.53)
Incomplete	0.98 (0.78–1.23)	0.86 (0.65–1.14)
Complete	1.00	1.00
Amniotic fluid		
Polyhydramnios	2.38 (1.26–4.50)	2.57 (0.98–6.75)
Oligohydramnios	0.87 (0.65–1.16)	0.88 (0.62–1.25)
Normal	1.00	1.00
Chorioamnionitis		
Yes	0.85 (0.69–1.05)	0.63 (0.49–0.81)
No	1.00	1.00
Multiple birth		
Yes	1.25 (1.00–1.54)	1.17 (0.90–1.53)
No	1.00	1.00

OR, odds ratio; CI, confidence interval; SGA, small for gestational age; AGA, appropriate for gestational age; PDA, patent ductus arteriosus.

^a^Results from the data with frequency matching by gestation and small for gestational age.

^b^Includes infants who received cardiac massage or were administered epinephrine.

### Yearly trend of hypotension medication

**Fig 2:**
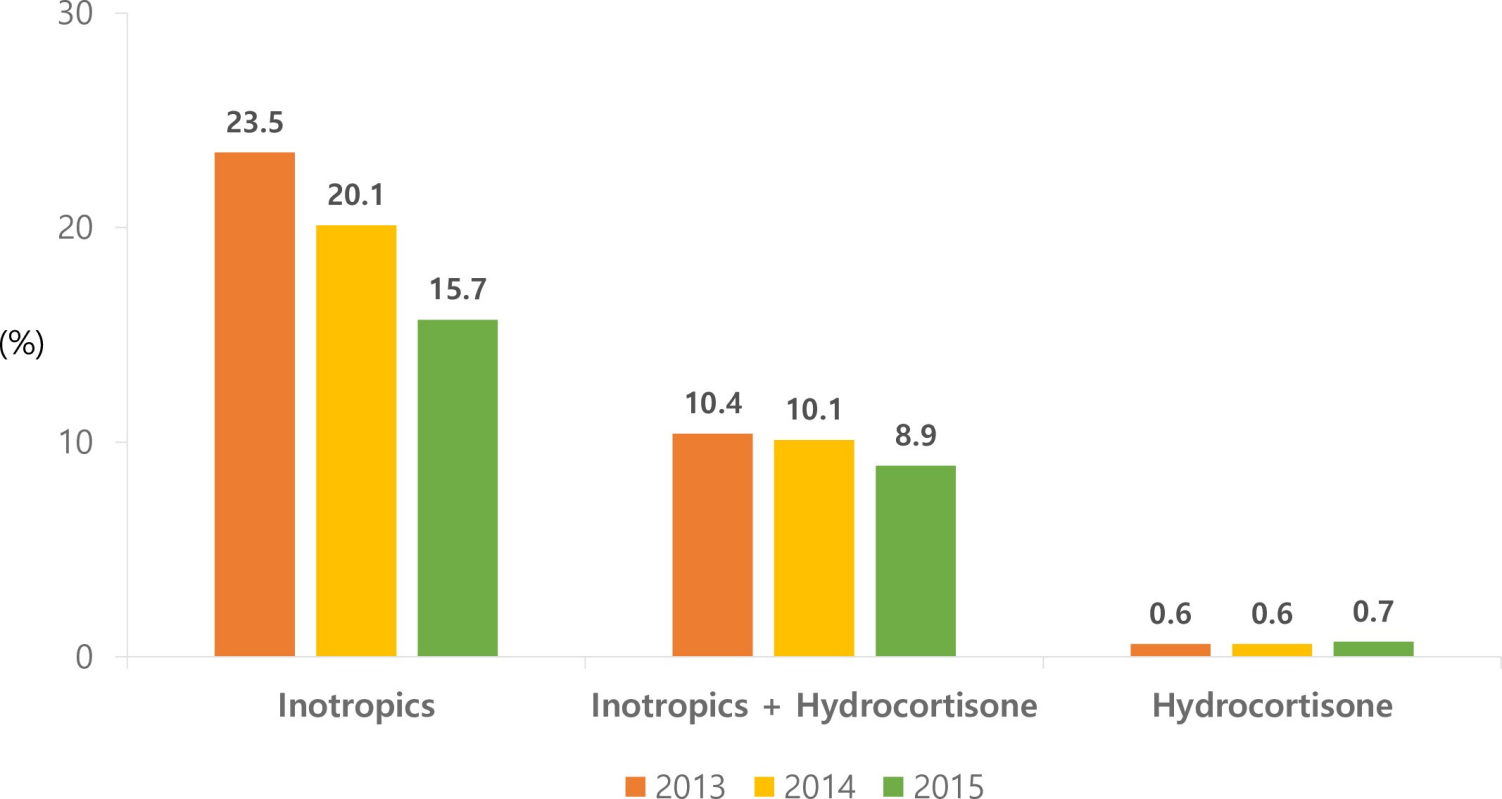
The proportion of the use of each hypotensive drug within the first postnatal week in VLBWI, 2013–2015.

Inotropic agents accounted for the highest proportion of hypotension treatment medications over the 3 years, followed by a combination therapy of inotropic agents and hydrocortisone (Fig 2). The trend of inotropics showed a statistically significant decrease for 3 years from 2013 to 2015 in the risk of occurrence of 0.817 (Confidence interval (CI) 0.749–0.891), and in the case of simultaneous administration of inotropics and hydrocortisone, the risk of occurrence was 0.925 (CI 0.817–1.046), which was not statistically significant.

### Comparison of prognosis at discharge of VLBW infants who were treated for hypotension during the first postnatal week

The mortality rate in the hypotension group was 43.2%, which was significantly higher than that in the control group (6.1%). Rates of grade 3 or higher IVH, PVL, moderate to severe BPD, and stage 3 or higher ROP were also significantly higher. Even after matching, the mortality rate, grade 3 or higher IVH, PVL, and moderate to severe BPD were still significantly higher in the hypotension group. Stage 3 or higher ROP did not differ significantly between the groups (Table 4).

**Table 4:** The morbidity of treated hypotension in VLBW infants during the first postnatal week.

					Matched population^a^		
Parameter		No hypotension (n = 2935)	Hypotension (n = 1256)	Total (n = 4191)	No hypotension (n = 1027)	Hypotension (n = 1027)	Total (n = 2054)
		n (%)	n (%)	n (%)	n (%)	n (%)	n (%)
Death		180 (6.1)	543 (43.2)*	723 (17.3)	129 (12.6)	383 (37.3)*	512 (24.9)
	within 24 h	24 (17.8)	29 (7.1)*	53 (9.7)	20 (19.8)	21 (7.3)*	41 (10.6)
	from 24 h to 7 days	59 (43.7)	208 (50.6)	267 (48.9)	41 (40.6)	139 (48.4)	180 (46.4)
	from 8 days to 28 days	52 (38.5)	174 (42.3)	226 (41.4)	39 (39.0)	127 (44.3)	166 (42.9)
IVH, ≥ grade 3		169 (5.9)	305 (26.9)*	474 (11.8)	107 (10.9)	227 (24.2)*	334 (17.4)
Periventricular leukomalacia		199 (6.9)	168 (15.1)*	367 (9.2)	77 (7.9)	151 (16.3)*	228 (11.9)
BPD, ≥ moderate		735 (26.5)	489 (64.0)*	1224 (34.6)	336 (36.9)	427 (62.3)*	763 (47.8)
ROP, ≥ stage 3		271 (9.8)	203 (25.7)*	474 (13.3)	186 (20.4)	162 (23.0)	348 (21.5)

IVH, intraventricular hemorrhage; BPD, brochopulmonary dysplasia; ROP, retinopathy of prematurity; VLBW, very low birth weight.

^a^Results from the data with frequency matching by gestation and small for gestational age.

*P < 0.01.

## Discussion

This study was a multi-center, nationwide, cohort study using the VLBW infant data provided by the KNN. Seventy hospitals currently participate in the KNN, which accounts for about 80% of NICUs in South Korea, and KNN data account for almost 70% of VLBW infants in South Korea.

**Fig 3:**
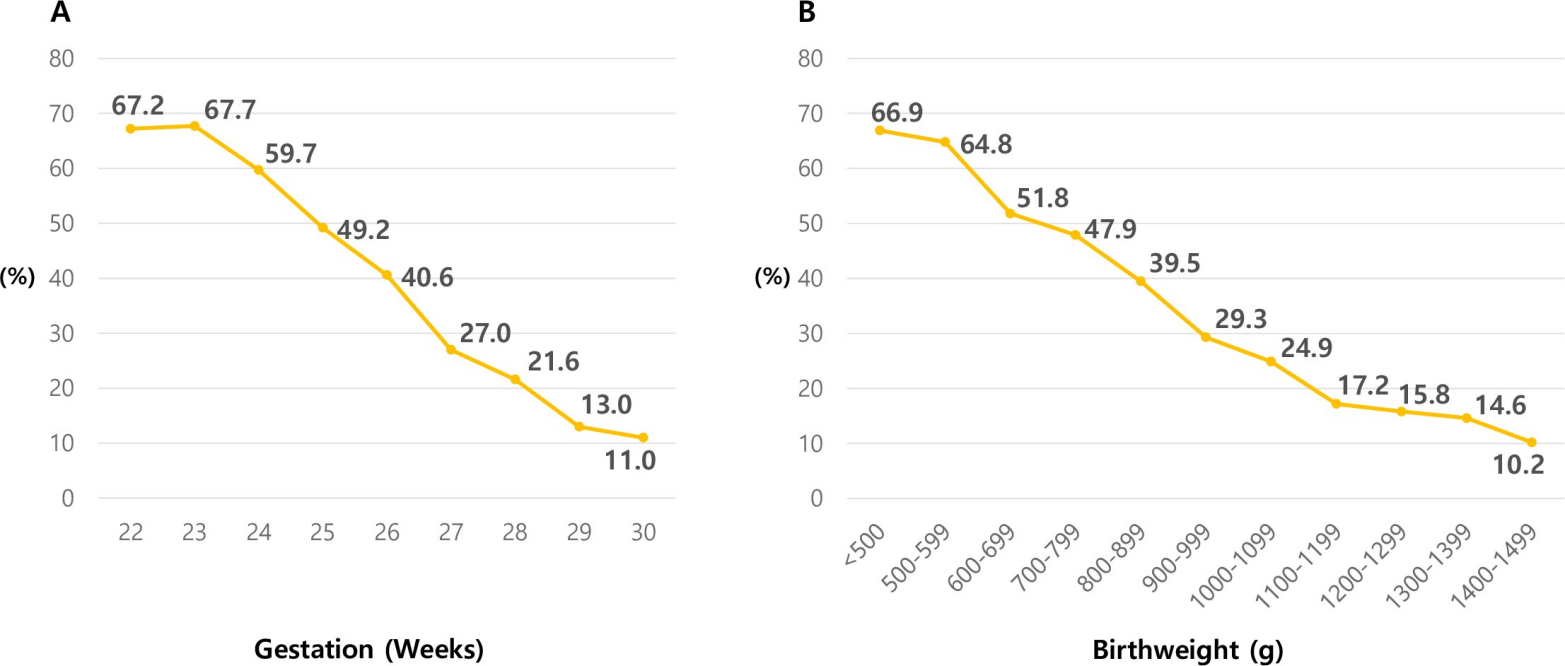
Changes in the proportion of treated hypotension in VLBW infants during the first postnatal week. (A) Changes according to gestation. (B) Changes according to birth weight.

Through these data, the incidence of hypotension according to gestational age, the rate was demonstrating a gradually decreasing pattern, and indicating a high correlation between gestational age and hypotension in preterm infants. The changes according to birth weight showed the same pattern (Fig 3).

In this study the gestational age, birth weight, and SGA status were identified as the most influential risk factors for hypotension as in the results of previous studies [5,8–10]. We identified the risk factors and prognosis of VLBW infants who treated for hypotension within one week after birth. To clarify this, the study was conducted using data excluding gestational age and SGA, which were found to be closely related to hypotension of VLBW infants.

Kim et al. reported that only intubation at delivery and epinephrine administration showed a significant correlation [18], and Hegyi et al. reported that in infants of the same gestational age, the rate of hypotension was high when a ventilator was applied or the Apgar score was low [13,24]. We also confirmed the 1-minute Apgar score, performing cardiac massage or administrating epinephrine at birth, symptomatic PDA, EOS, and maternal chorioamnionitis as factors associated with the need for treatment for neonatal hypotension. In particular, the 1-minute Apgar score and administration of resuscitation were significantly associated with neonatal hypotension early after birth, and the results were consistent before and after matching. The neonatal state and early intervention after birth are crucial to reduce the incidence of neonatal hypotension in preterm infants, therefore proper prenatal examination, proper obstetrics and gynecology care, and skilled neonatal resuscitation are necessary.

Previous studies reported that when the mother had chorioamnionitis, interleukin-6 and interleukin-1β in cord blood increased to relax the vascular smooth muscle, causing tachycardia and hypotension in the newborn [12,25]. However, recent reports show that hypotension may occur as a complication of preterm birth due to chorioamnionitis rather than chorioamnionitis itself or that chorioamnionitis itself is not significantly associated with early neonatal hypotension [5,26]. In this study, chorioamnionitis and treated hypotension did not show a statistically significant association before matching. However, the incidence of hypotension was significantly lower in infants whose mothers had chorioamnionitis than in infants of mothers not having chorioamnionitis after matching. And it was found that the rate of chorioamnionitis is higher as the gestational age is lower. Additionally, in the 22–24 weeks group, there was no statistical significance between the incidence of hypotension and chorioamnionitis before and after matching. This analysis is meaningful because it is obtained by matching the gestational age and studying only the 22–24 week group to analyze the association between chorioamnionitis and treated hypotension.

We further analyzed the relationship between sepsis and the hypotension in mothers with chorioamnionitis. The proportion of the hypotension group with sepsis was higher than that without sepsis regardless of maternal chorioamnionitis. The same result was obtained based on the data after matching. It seemed that sepsis, rather than maternal chorioamnionitis was associated with hypotension.

Among prenatal factors, prenatal steroid administration to the mother is also known to lower the rate of hypotension in preterm infants [7,27], but the related studies did not completely exclude the effect of gestational age. Another study reported less of an association between prenatal steroid administration and hypotension when groups with similar gestational ages were compared [8]. In our study, the rate of hypotension was high when prenatal steroids were not used in simple comparison, and in the regression analysis, prenatal steroids showed a tendency to be associated with hypotension, but this was not statistically significant before and after matching.

Maternal polyhydramnios is known to be associated with infection or congenital anomalies, thus increasing neonatal mortality and morbidity [28]. In this study, maternal polyhydramnios was associated with hypotension early after birth, but the statistical significance was lost after matching. However, maternal polyhydramnios has been reported to be an independent predictor of mortality or morbidity [29], and subsequent studies will be needed with using a larger number of VLBW infants with maternal polyhydramnios to address the small sample size in this study.

IVH of grade 3 or higher, PVL, and moderate to severe BPD showed significantly higher prevalence in hypotensive infants receiving treatment both before and after matching in the hypotension group. Previous studies have reported a higher incidence of mortality and neurological complications, such as IVH and PVL, in infants with neonatal hypotension [30–32], and although there were limitations such as small subject numbers, there have been reports of no association between diseases such as ROP, NEC, and BPD, other than neurological complications and hypotension [16,33]. Our study involved 1,256 patients treated with hypotension within the first postnatal week, and this represents a large number of subjects, and as the effects of gestational age and birth weight were excluded, the results of this study can be regarded as more specific results. The rate of stage 3 or higher ROP showed no statistical difference after excluding the effects of gestational age and birth weight, and it is thought that stage 3 or higher ROP is more associated with gestational age or birth weight than with neonatal hypotension.

Based on our study data, inotropic agents were the most common drugs used to treat hypotension in VLBW infants (63.6%), followed by a combination of inotropic agents and hydrocortisone, which accounted for 32.2%. Hydrocortisone monotherapy accounted for only 2.1% of the total. In a study conducted by Stranak et al. on extremely low gestational age newborn’s hypotension treatment for NICUs in 38 EU countries, dopamine was the most frequently selected drug for hypotension treatment, and additional drugs showed different rates of administration among hospitals [34]. Another study of NICUs in Canada reported that the rate of infants who received a combination therapy of dopamine and dobutamine or dopamine and hydrocortisone was similar [35]. The decreasing trend of the proportion of inotropics alone administered in this study is thought to be related to the NICU treatment tendency to allow permissive hypotension and hydrocortisone administration, which is associated with adrenal insufficiency in early prematurity [36].

We additionally examined the risk factors of hypotension in the infants born at 22–24 weeks of gestation and were treated within a week after birth. Gestational age and birth weight were not significantly different before and after matching in this group. The identified risk factors were consistently similar to findings for the whole VLBW infants. In the results of logistic regression, which confirmed the association with hypotension, the incidence of hypotension was increased in these infants with 1-minute Apgar score ≤ 3, who underwent neonatal resuscitation, or diagnosed EOS. Symptomatic PDA, maternal polyhydramnios, and chorioamnionitis disappeared from the association with hypotension. Compared to the results of entire VLBW infant group, the 22–24 week group showed similarly high rate of symptomatic PDA in both the hypotension group and the control group, so there seems to be no statistical significance (S1–S4 Tables). This result is thought to be because the 22–24 week group is very immature and hemodynamically unstable.

A standardized definition of hypotension has not yet been established. In addition to the definition used in this study, hypotension in preterm infants is sometimes defined as a MAP < 5th percentile based on the MAP distribution of preterm infants of the same gestational age [37]. Further, treatment for hypotension may not be determined solely by numerical values, but is also influenced by the cause of hypotension and the accompanying clinical symptoms (abnormal blood circulation, decreased cerebral blood flow), and whether shock progresses. [38,39]. Therefore, the subjects of this study were limited to those who showed hypotension within 7 days of age and were treated with hypotensive drugs.

This study has the following limitations. First, we have not been able to collect data on when treatment for hypotension was started. Second, we do not have data on how often low blood pressure occurred but was managed as permissive hypotension and not treated pharmacologically. Permissive hypotension is performed in most NICUs in Korea, and studies on the results of permissive hypotension per unit have also been published [4]. However, since the KNN data used in this study are national multicenter neonatal network based on a prospective web-based registry of VLBWIs hospitalized at 70 NICUs in Korea, specific details or diagnostic criteria on permissive hypotension were not available.

Nevertheless, our study is meaningful in that it used data on more than 80% of VLBWIs in Korea at the time of their admission to the NICU and after discharge. In addition, we believe that matching the gestational age and SGA to exclude the effects of birth weight and gestational age, which are known to affect hypotension of VLBWIs, was useful in obtaining more accurate results.

In conclusion, through this study, we were able to confirm that hypotension treated within one week of life was associated with the 1-minute Apgar score, performing cardiac massage or administrating epinephrine at birth, symptomatic PDA, EOS, and maternal chorioamnionitis. In particular, it was found that factors that indicate the initial state of premature infants, such as the 1-minute Apgar score or performing neonatal resuscitation, are closely related to hypotension within one week after birth. Therefore, to reduce the occurrence of hypotension of early stage of life in premature infants, regular prenatal care, including careful monitoring, and proper neonatal resuscitation at birth are needed.

## Supporting information

S1 Table Demographic characteristics of populations (22–24 weeks). (DOCX)

S2 Table Risk factors of treated hypotension in VLBW infants during the first postnatal week (22–24 weeks). (DOCX)

S3 Table Multivariate logistic regression model for risk factors (22–24 weeks). (DOCX)

S4 Table The morbidity of treated hypotension in VLBW infants during the first postnatal week (22–24 weeks). (DOCX)
