# Supplementary material for: Risk factors and prognosis in very low birth weight infants treated for hypotension during the first postnatal week from the Korean Neonatal Network
Source: PLoS One. 2021 Oct 14;16(10):e0258328. doi: 10.1371/journal.pone.0258328 (PMC8516276; doi:10.1371/journal.pone.0258328)
Supplement: S1 Table — (DOCX) [file pone.0258328.s001.docx]

|  | |  |  |  |  |  | **Matched populations^a^** | | |
| --- | --- | --- | --- | --- | --- | --- | --- | --- | --- |
| **Parameters** | |  | **No hypotension**  **(n=200)** | **Hypotension**  **(n=346)** | **Total**  **(n=546)** |  | **No hypotension**  **(n=192)** | **Hypotension**  **(n=192)** | **Total**  **(n=384)** |
|  |  |  | **n (%)** | **n (%)** | **n (%)** |  | **n (%)** | **n (%)** | **n (%)** |
| **Neonatal** | |  |  |  |  |  |  |  |  |
| Gestational age (weeks, mean±SD) | |  | 23.5±0.7 | 23.4±0.7 | 23.4±0.7 |  | 23.5±0.6 | 23.5±0.6 | 23.5±0.6 |
| Gestational age, | 22 weeks |  | 20 (32.8) | 41 (67.2) | 61 (100.0) |  | 16 (51.6) | 16 (48.4) | 32 (100.0) |
|  | 23 weeks |  | 63 (32.3) | 132 (67.7) | 195 (100.0) |  | 63 (50.0) | 63 (50.0) | 126 (100.0) |
|  | 24 weeks |  | 117 (40.3) | 173 (59.7) | 290 (100.0) |  | 113 (50.0) | 113 (50.0) | 226 (100.0) |
| Birth weight (g, mean±SD) | |  | 641.5±117 | 626.5±126 | 632.0±123 |  | 645.6±116 | 648.2±118 | 646.9±117 |
| Birth weight, | < 500 g |  | 22 (31.4) | 48 (68.6) | 70 (100.0) |  | 19 (48.7) | 20 (51.3) | 39 (100.0) |
|  | 500–999 g |  | 178 (37.5) | 297 (62.5) | 475 (100.0) |  | 173 (50.3) | 172 (49.7) | 345 (100.0) |
|  | 1000–1499 g |  | 0 (0.0) | 1 (100.0) | 1 (100.0) |  | 0 (0.0) | 0 (0.0) | 0 (0.0) |
| Small for gestational age < 3^rd^ percentile | |  | 7 (25.0) | 21 (75.0) | 28 (100.0) |  | 7 (50.0) | 7 (50.0) | 14 (100.0) |
|  | 3^–^9^th^ percentile |  | 8 (34.8) | 15 (65.2) | 23 (100.0) |  | 7 (50.0) | 7 (50.0) | 14 (100.0) |
| Appropriate for gestational age or above | |  | 174 (36.6) | 302 (63.4) | 476 (100.0) |  | 170 (50.0) | 170 (50.0) | 340 (100.0) |
| Sex ratio | |  | 48.5:51.5 | 49.7:50.3 | 49.3:50.7 |  | 49.0:51.0 | 50.5:49.5 | 49.7:50.3 |
| **Maternal** | |  |  |  |  |  |  |  |  |
| Age (years, mean±SD) | |  | 32.9±3.7 | 32.6±4.2 | 32.7±4.0 |  | 32.9±3.7 | 32.6±4.4 | 32.8±4.1 |
| Pregnancy process, IVF | |  | 55 (27.5) | 89 (25.7) | 144 (26.4) |  | 53 (27.6) | 45 (23.4) | 98 (25.5) |
| Delivery by cesarean section | |  | 128 (64.0) | 206 (59.5) | 334 (61.2) |  | 125 (65.1) | 118 (61.5) | 243 (63.3) |

**S1 Table. Demographic characteristics of populations (22-24 weeks)**

SD, standard deviation; IVF, in vitro fertilization.

^a^Results from the data with frequency matching by gestation and small for gestational age.

^*^***P*** < 0.01, ^**^***P*** < 0.05
