# Supplementary material for: Risk factors and prognosis in very low birth weight infants treated for hypotension during the first postnatal week from the Korean Neonatal Network
Source: PLoS One. 2021 Oct 14;16(10):e0258328. doi: 10.1371/journal.pone.0258328 (PMC8516276; doi:10.1371/journal.pone.0258328)
Supplement: S2 Table — (DOCX) [file pone.0258328.s002.docx]

**S2 Table. Risk factors of treated hypotension in VLBW infants during the first postnatal week (22-24 weeks)**

|  |  |  |  |  |  | **Matched population^a^** | | |
| --- | --- | --- | --- | --- | --- | --- | --- | --- |
| **Parameters** |  | **No hypotension**  **(n=200)** | **Hypotension**  **(n=346)** | **Total**  **(n=546)** |  | **No hypotension**  **(n=192)** | **Hypotension**  **(n=192)** | **Total**  **(n=384)** |
|  |  | **n (%)** | **n (%)** | **n (%)** |  | **n (%)** | **n (%)** | **n (%)** |
| **Neonatal** |  |  |  |  |  |  |  |  |
| Apgar score at 1min (Mean±SD) |  | 3.0±1.6 | 2.5±1.6^*^ | 2.7±1.6 |  | 3.0±1.6 | 2.6±1.6^*^ | 2.8±1.6 |
| Apgar score at 1min, 0-3 |  | 124 (62.6) | 259 (75.1)^*^ | 383 (70.5) |  | 119 (62.6) | 140 (72.9)^**^ | 259 (67.8) |
| Apgar score at 5min (Mean±SD) |  | 5.3±2.0 | 4.8±2.0^*^ | 5.0±2.0 |  | 5.4±2.0 | 48±2.1^*^ | 5.1±2.0 |
| Apgar score at 5min, 0-3 |  | 41 (20.7) | 91 (26.4) | 132 (24.3) |  | 38 (20.0) | 50 (26.0) | 88 (23.0) |
| Cardiac massage |  | 17 (8.5) | 62 (18.1)^*^ | 79 (14.6) |  | 16 (8.4) | 38 (20.0)^*^ | 54 (14.2) |
| Epinephrine administration |  | 12 (6.0) | 44 (12.8)^**^ | 56 (10.3) |  | 11 (5.8) | 25 (13.2)^**^ | 36 (9.4) |
| Initial BT, <36℃ |  | 93 (51.1) | 160 (50.0) | 253 (50.4) |  | 84 (48.0) | 88 (48.9) | 172 (48.5) |
| Initial pH, <7.20 |  | 39 (28.5) | 101 (41.2)^**^ | 140 (36.6) |  | 39 (29.1) | 54 (42.9)^**^ | 93 (35.8) |
| CRIB-II score (Mean±SD) |  | 12.9±1.3 | 13.1±1.4 | 13.0±1.4 |  | 12.9 ±1.3 | 12.9±1.4 | 12.9±1.4 |
| CRIB-II score, ≥ 11 points |  | 194 (97.0) | 337 (97.4) | 531 (97.3) |  | 125 (99.2) | 120 (97.6) | 245 (98.4) |
| Symptomatic PDA |  | 102 (51.0) | 174 (50.3) | 276 (50.5) |  | 101 (54.9) | 98 (51.0) | 199 (52.9) |
| Early onset sepsis |  | 12 (6.0) | 44 (12.7)^**^ | 56 (10.3) |  | 12 (6.3) | 28 (14.6)^*^ | 40 (10.4) |
| **Maternal** |  |  |  |  |  |  |  |  |
| Antenatal steroid |  |  |  |  |  |  |  |  |
| None |  | 48 (24.2) | 100 (29.8) | 148 (27.7) |  | 45 (23.7) | 56 (30.3)^**^ | 101 (26.9) |
| Incomplete |  | 63 (31.8) | 114 (33.9) | 177 (33.1) |  | 61 (32.1) | 71 (38.4)^**^ | 132 (35.2) |
| Complete |  | 87 (43.9) | 122 (36.3) | 209 (39.1) |  | 84 (44.2) | 58 (31.4)^**^ | 142 (37.9) |
| Polyhydramnios |  | 2 (1.1) | 12 (3.9) | 14 (2.9) |  | 2 (1.2) | 5 (2.9) | 7 (2.1) |
| Oligohydraminos |  | 27 (15.4) | 53 (17.3) | 80 (16.6) |  | 26 (15.3) | 28 (16.6) | 54 (15.9) |
| Multiple birth |  | 71 (35.5) | 133 (38.4) | 204 (37.4) |  | 68 (35.4) | 69 (35.9) | 137 (35.7) |
| PROM |  | 94 (47.0) | 176 (51.3) | 270 (49.7) |  | 92 (47.9) | 104 (54.2) | 196 (51.0) |
| Chorioamnionitis |  | 97 (58.1) | 156 (53.6) | 253 (55.2) |  | 92 (57.5) | 92 (57.1) | 184 (57.3) |
| DM^b^ |  | 3 (1.5) | 14 (4.0) | 17 (3.1) |  | 3 (1.6) | 12 (6.3)^**^ | 15 (3.9) |
| HTN^c^ |  | 9 (4.5) | 23 (6.6) | 32 (5.9) |  | 8 (4.2) | 11 (5.7) | 19 (4.9) |

SD, standard deviation; BT, body temperature; CRIB, critical risk index for babies; PDA, patent ductus arteriosus; PROM, premature rupture of membrane; DM, diabetes mellitus; HTN, hypertension; VLBW, very low birth weight.

^a^Results from the data with frequency matching by gestation and small for gestational age.

^b^DM included gestational and overt diabetes mellitus.

^c^HTN included pregnancy induced hypertension and chronic hypertension.

^*^***P*** < 0.01; ^**^***P*** < 0.05.
