# Supplementary material for: Risk factors and prognosis in very low birth weight infants treated for hypotension during the first postnatal week from the Korean Neonatal Network
Source: PLoS One. 2021 Oct 14;16(10):e0258328. doi: 10.1371/journal.pone.0258328 (PMC8516276; doi:10.1371/journal.pone.0258328)
Supplement: S3 Table — (DOCX) [file pone.0258328.s003.docx]

**S3 Table. Multivariate logistic regression model for risk factors (22-24 weeks)**

| **Risk factors** |  |  |  | **Matched population^a^** |
| --- | --- | --- | --- | --- |
|  |  | **Adjusted OR (95% CI)** |  | **Adjusted OR (95% CI)** |
| Birth weight |  |  |  |  |
| < 500 g |  | 0.66 (0.17-2.61) |  | 0.69 (0.21-2.25) |
| 500-999 g |  | - |  | - |
| 1000-1499 g |  | 1.00 |  | 1.00 |
| SGA |  |  |  |  |
| < 3%ile |  | 4.95 (0.74-33.29) |  | - |
| 3-9%ile |  | 0.89 (0.19-4.12) |  | - |
| AGA or above |  | 1.00 |  | - |
| Apgar score 1min, 0-3 |  |  |  |  |
| Yes |  | 2.27 (1.30-3.97) |  | 1.84 (0.94-3.63) |
| No |  | 1.00 |  | 1.00 |
| Neonatal resuscitation^b^ |  |  |  |  |
| Yes |  | 3.22 (1.13-9.23) |  | 3.70 (1.19-11.48) |
| No |  | 1.00 |  | 1.00 |
| Body temperature at admission |  |  |  |  |
| < 36℃ |  | 0.67 (0.39-1.15) |  | 0.58 (0.31-1.09) |
| ≥ 36℃ |  | 1.00 |  | 1.00 |
| Initial pH at admission |  |  |  |  |
| < 7.20 |  | 1.48 (0.83-2.64) |  | 1.27 (0.65-2.48) |
| ≥ 7.20 |  | 1.00 |  | 1.00 |
| Symptomatic PDA |  |  |  |  |
| Yes |  | 1.00 (0.58-1.73) |  | 0.94 (0.49-1.79) |
| No |  | 1.00 |  | 1.00 |
| Early onset sepsis |  |  |  |  |
| Yes |  | 2.35 (0.96-5.74) |  | 2.72 (1.06-7.01) |
| No |  | 1.00 |  | 1.00 |
| Antenatal steroid |  |  |  |  |
| None |  | 2.26 (1.09-4.69) |  | 2.26 (0.98-5.22) |
| Incomplete |  | 1.39 (0.77-2.52) |  | 1.48 (0.74-2.96) |
| Complete |  | 1.00 |  | 1.00 |
| Amniotic fluid |  |  |  |  |
| Polyhydramnios |  | 1.62 (0.27-9.64) |  | 1.48 (0.22-9.76) |
| Oligohydramnios |  | 1.53 (0.73-3.20) |  | 1.19 (0.49-2.88) |
| Normal |  | 1.00 |  | 1.00 |
| Chorioamnionitis |  |  |  |  |
| Yes |  | 0.70 (0.41-1.21) |  | 0.65 (0.35-1.24) |
| No |  | 1.00 |  | 1.00 |
| Multiple birth |  |  |  |  |
| Yes |  | 1.14 (0.66-1.99) |  | 0.74 (0.38-1.44) |
| No |  | 1.00 |  | 1.00 |

OR, odds ratio; CI, confidence interval; SGA, small for gestational age; AGA, appropriate for gestational age; PDA, patent ductus arteriosus.

^a^Results from the data with frequency matching by gestation and small for gestational age.

^b^Include infants who were conducted cardiac massage or administered epinephrine.
