# Supplementary material for: Risk factors and prognosis in very low birth weight infants treated for hypotension during the first postnatal week from the Korean Neonatal Network
Source: PLoS One. 2021 Oct 14;16(10):e0258328. doi: 10.1371/journal.pone.0258328 (PMC8516276; doi:10.1371/journal.pone.0258328)
Supplement: S4 Table — (DOCX) [file pone.0258328.s004.docx]

**S4 Table. The Morbidity of treated hypotension in VLBW infants during the first postnatal week (22-24 weeks)**

|  | |  |  |  |  |  | **Matched population^a^** | | |
| --- | --- | --- | --- | --- | --- | --- | --- | --- | --- |
| **Parameters** | |  | **No hypotension**  **(n=200)** | **Hypotension**  **(n=346)** | **Total**  **(n=546)** |  | **No hypotension**  **(n=192)** | **Hypotension**  **(n=192)** | **Total**  **(n=384)** |
|  |  |  | **n (%)** | **n (%)** | **n (%)** |  | **n (%)** | **n (%)** | **n (%)** |
| Death | |  |  |  |  |  |  |  |  |
|  | within 24hrs |  | 18 (29.5) | 10 (5.3)^*^ | 28 (11.3) |  | 16 (27.6) | 6 (5.8)^*^ | 22 (13.7) |
|  | from 24hrs to 7 days |  | 21 (34.4) | 102 (54.5)^*^ | 123 (49.6) |  | 20 (34.5) | 52 (50.5)^**^ | 72 (44.7) |
|  | from 8 days to 28 days |  | 22 (36.1) | 75 (40.1) | 97 (39.1) |  | 22 (37.9) | 45 (43.7) | 67 (41.6) |
| IVH, ≥ grade 3 | |  | 43 (25.4) | 133 (45.4)^*^ | 176 (38.1) |  | 41 (25.0) | 71 (43.0)^*^ | 112 (34.0) |
| Periventricular leukomalacia | |  | 25 (14.9) | 31 (10.9) | 56 (12.4) |  | 23 (14.1) | 20 (12.4) | 43 (13.3) |
| BPD, ≥ moderate | |  | 81 (62.8) | 107 (84.3)^*^ | 188 (73.4) |  | 78 (61.9) | 67 (85.9)^*^ | 145 (71.1) |
| ROP, ≥ stage 3 | |  | 70 (54.3) | 70 (52.6) | 140 (53.4) |  | 68 (54.0) | 39 (48.1) | 107 (51.7) |

IVH, intraventricular hemorrhage; BPD, brochopulmonary dysplasia; ROP, retinopathy of prematurity; VLBW, very low birth weight.

^a^Results from the data with frequency matching by gestation and small for gestational age.

^*^***P*** < 0.01; ^**^***P*** < 0.05.
